# Impact of Formal and Lifelong Education on Croatian Nurses’ Knowledge of Pressure Ulcers: A Cross-Sectional Study

**DOI:** 10.3390/nursrep15110407

**Published:** 2025-11-18

**Authors:** Ana Žepina Puzić, Mirna Žulec, Vesna Bušac, Želimir Bertić, Bojana Filej

**Affiliations:** 1 Department of Health Studies, Šibenik University of Applied Sciences, 22000 Šibenik, Croatia; vesna.busac@vus.hr; 2 Faculty of Health Sciences, University of Novo Mesto, 8000 Novo Mesto, Slovenia; bojana.filej@guest.arnes.si; 3 Department of Nursing, Catholic University of Croatia, 10000 Zagreb, Croatia; 4 Department of Health Studies, University of Applied Sciences Ivanić Grad, 10300 Ivanić Grad, Croatia; 5 Faculty of Health Studies, University of Mostar, 88000 Mostar, Bosnia and Herzegovina; 6 Department of Nursing, Faculty of Health Studies, International University of Rijeka, 51000 Rijeka, Croatia; 7 Institute of Public Health of Bjelovar-Bilogora County, 43000 Bjelovar, Croatia

**Keywords:** nursing curriculum, Pieper-Zulkowski Pressure Ulcer Knowledge Test, healthcare, nursing, knowledge

## Abstract

**Background/Objectives**: Formal and lifelong education on pressure injuries enables the acquisition and maintenance of relevant knowledge and represents a key link in ensuring quality and safe care. Critical planning and continuous implementation of education programs directly contributes to better management of these injuries and ensures consistent application of best practices in everyday nursing care. **Methods**: This study is based on a cross-sectional, quantitative methodology conducted in a state hospital in Croatia, on a sample of 268 participants. **Results**: Work experience did not show a significant correlation with test scores while the level of education, a master’s degree in nursing, had the highest mean percentages in prevention and overall score. Differences in test performance across department type showed a significant difference for the prevention subscale for neurological department staff. Activities such as reading the professional literature and attending lectures showed a positive correlation with higher scores in different areas of prevention, staging and the overall test. Internet use was positively associated with all subscales and the overall score. **Conclusions:** Despite the unsatisfactory level of knowledge of the respondents, the positive impact of formal and lifelong education on the existing level of information about pressure ulcers is evident. These findings highlight the need for improvement and better organization of educational programs, with an emphasis on their accessibility, continuity and focus on clearly defined learning outcomes.

## 1. Introduction

### 1.1. Definition and Impact of Pressure Ulcers/Injuries (PUs/PIs)

A pressure ulcer/injury (PU/PI) is a localized injury to the skin and/or tissue [1], usually at the site of a bony prominence, resulting from pressure or pressure combined with shear [2], but it can also be associated with a medical device or other object [3]. It is a serious, painful and disabling condition for people of all ages, significantly impairing quality of life and requiring evidence-based prevention and management of pressure injuries [4]. Understanding prevention and management is a fundamental component of nursing practice aimed at ensuring safe and high-quality patient care [5]. Nurses, as key health professionals, play a leading role in prevention and treatment [6]. In their daily work, nurses encounter various barriers to providing evidence-based and effective care, such as limited time, workload pressures, and insufficient institutional support. Consequently, attaining sufficient knowledge through both formal and continuing education, along with adequate institutional support, is essential for the effective prevention and management of these injuries [7,8].

### 1.2. Importance of Evidence-Based Knowledge

The effective implementation of evidence-based prevention strategies is often hindered by a lack of adequate knowledge and practical skills [9]. The education that nurses receive today shapes and influences the nurses of the future [10]. Effective education and professional training in prevention and treatment enhance the abilities of healthcare providers [11].

### 1.3. Challenges in Formal Education

In nursing education at secondary and undergraduate levels, this issue is addressed within the core subjects defined by the curriculum [12]. However, the curriculum only contains a list of core thematic units without a precisely defined number of hours for their treatment, which leaves teachers with freedom in planning and delivering lessons. As long as an insufficient level of knowledge is recorded, a significant decrease in the prevalence of PU/PI cannot be expected. This highlights the need to improve the part of the curriculum on the topic within formal education [13].

The relationship between continuous education and nurses in terms of thematic areas emphasizes that the problem also arises from the literature search and decisions made based on current information [14].

### 1.4. Limitations of Continuing Education

Since systematic education is generally not available, education on the prevention and treatment of PU/PI in Croatia as part of lifelong learning depends on the interest of the healthcare institution and the individual motivation of nurses. Also, after completing the education program, there is no assessment on the acquired knowledge [15]. Previous research has mainly focused on the level of knowledge of nurses or on the effectiveness of individual educational interventions, while the impact of different forms of education (formal and lifelong) on the knowledge and application of acquired skills remains insufficiently investigated. Therefore, this study aims to fill a gap in the literature on comparing the effectiveness of formal and informal educational approaches in everyday nursing practices.

## 2. Materials and Methods

This study employed a cross-sectional design and was conducted in a public hospital in Croatia. Its primary objective was to assess the level of knowledge that nurses possess about pressure ulcers and injuries, with special attention given to how formal education and lifelong learning contribute to their understanding of the issue. The research further aimed to explore the extent to which various educational pathways—such as academic programs, professional development courses, and ongoing training initiatives—affect nurses’ theoretical and practical competence in the prevention, description and staging of PU/PI. This study also sought to identify key determinants contributing to differences in knowledge among nurses and provide guidelines for improving educational strategies to enhance the quality of care and patient outcomes. Results were reported in accordance with STROBE guidelines for observational studies.

### 2.1. Measurement Instrument

The study employed the Croatian version of the Pieper-Zulkowski Pressure Ulcer Knowledge Test Version 2 (PZ-PUKT), a two-part instrument for assessing knowledge. The effectiveness of the PZ-PUKT tool for measuring nurses’ knowledge of PU/PI was also determined through a systematic literature review [16]. The first part of the questionnaire consists of 11 sociodemographic questions and items regarding the sources and methods of knowledge acquisition, while the second part includes 72 items of PZ-PUKT. The items are divided into three subscales: Prevention (31 items), Wound Description (20 items) and Staging (21 items). Responses to each item can be “True”, “False” or “Don’t know”, and the total range of points of the instrument is 72. The Croatian version of PZ-PUKT has an overall KR-20 of 0.79, Guttman Lambda 6 of 0.89, and overall test–retest reliability (ICC) of 0.91 [17].

### 2.2. Data Collection

Data collection took place using a paper-and-pencil version of the questionnaire between March and May 2024. All participants were informed about the purpose and objectives of the study and signed an informed consent form. Questionnaire completion was anonymous and voluntary and required about 20 min. Only completely answered questionnaires were processed.

### 2.3. Sample

The research was conducted in a state hospital in Croatia with 20 hospital departments, 257 beds, and 497 employed nurses of all levels of education. Based on the number of employed nurses, the optimal sample size for this research was determined according to Horvat and Mioč [18]: using a confidence level of 95% with a margin of error of 5%, the sample required was determined to be n = 217 respondents. In total, 300 questionnaires were distributed, and after excluding incomplete questionnaires for processing, 268 respondents remained for analysis with a response rate of 89.33%.

### 2.4. Data Analysis

Descriptive statistics were employed to summarize the sample characteristics, including means and standard deviations for continuous variables and frequencies with percentages for categorical variables. Initially, the assumption of normality of all continuous variables was checked using the Shapiro–Wilk W test and QQ plots. Additionally, the assumptions for ANOVA, namely, normality of residuals, homogeneity of variances and lack of any influential outliers, were also checked before analysis interpretation.

Differences in test performance across education levels and department types were examined using one-way analysis of variance (ANOVA). When a significant main effect was detected, post hoc Tukey’s Honestly Significant Difference (HSD) tests were conducted to identify pairwise differences between groups.

To assess relationships between variables, Spearman’s rank-order correlation was employed to evaluate the associations between educational exposure (lecture attendance and reading) and test performance in Prevention, Staging, Wound Description and overall scores.

Independent samples *t*-tests were conducted to compare test performance between groups based on internet usage for pressure ulcer information. Differences in Prevention, Staging, Wound Description and overall scores were examined between participants who reported using the internet for this purpose and those who did not. The strength of the correlation was interpreted according to Cohen’s classification, with which correlation coefficient values are considered weak up to 0.10, moderate around 0.30, and strong at 0.50 and higher [19]. Statistical significance was set at *p* < 0.05, and all statistical analyses were conducted using R software (version 4.3.1 Foundation for Statistical Computing, Vienna, Austria) and Jamovi (2.3.28.0 version 2.3.28.0; jamovi Project, Sydney, Australia).

### 2.5. Research Ethics

The research was conducted in accordance with the ethical standards of the Declaration of Helsinki (version 2013). The research participants were guaranteed anonymity and confidentiality. In accordance with ethical principles, the respondents were presented with the details of the study before consenting to participate. At the very beginning, respondents were informed about the purpose, goals and manner in which their data will be employed. Participation in the research did not entail any risks. Participants had the option to withdraw from the study at any time. The research was conducted using a printed version of the questionnaire. Before conducting the research, permission to conduct the study was given by the Ethics Committee of the Šibenik-Knin County General Hospital (Class: 007-10/24-01/3, Reg. no.: 2182-1-50-01-01-24-1).

## 3. Results

### 3.1. Basic Sociodemographic Characteristics of the Respondents

Table 1 presents the sample consisting of 268 participants across all education levels, with the majority being female (97.4%). The mean age varied by group, with technicians averaging 39.2 years; bachelor’s degree holders, 35.1 years; and master’s degree holders, 42.8 years. Work experience was highest among master’s degree holders (17.7 years) and technicians (17.3 years), while holders of bachelor’s degrees had the least experience (12.3 years). Analysis of the distribution of respondents by workplace revealed that the largest proportion was made up of nurses employed in surgical departments (28.7%). This was followed by nurses from internal medicine departments (21.6%), and then neurological departments (20.9%) and intensive care units (8.2%). Other departments were represented with a share of 20.5%.

### 3.2. Basic Descriptive Sample Parameters by Education Level

Educational engagement varied, with 27.6% of participants attending lectures for 2–3 years and 41.4% reporting they had read about the topic for less than a year. Internet use for education was highest among bachelor’s degree holders (60.8%) and lowest among technicians (37.2%). Master’s degree holders had the highest mean percentages in Prevention (67.0%), Wound Description (58.6%) and overall score (61.1%), while bachelor’s degree holders had the highest score in Staging (59.0%). These results are presented in Table 2.

### 3.3. Test Percentages by Education Level

One-way ANOVA was used to examine differences in test performance across education levels (technician, bachelor’s, master’s) for Prevention, Staging, Wound Description and overall percentages. These results are presented in Table 3.

### 3.4. Test Percentages by Department

Table 4 shows the results of a one-way ANOVA conducted to examine differences in test performance percentages across department types (Surgery, Internal, Neurology, ICU and Other).

### 3.5. Correlation of Lecture and Read with Test Percentages

Spearman’s correlation analysis examined the relationships between the recency of attending a lecture (Lecture) and reading about pressure ulcers (Read) with test performance (Prevention, Staging, Wound Description and overall scores). These results are presented in Table 5.

### 3.6. Test Percentages by Use of Internet for PU/PI Information

The independent samples *t*-test examined differences in test performance percentages between participants who employed the internet for information on pressure ulcers (Yes) and those who did not (No). These results are presented in Table 6.

## 4. Discussion

The results of this study show that the level of knowledge of nurses was relatively low, with the weakest results recorded for the Wound Description and Staging subscales (54.7%, 56.1%). Also, in the Prevention subscale, the results (65.7%) were significantly higher compared to the others, although still insufficient, which indicates certain differences in acquired knowledge. The overall result of the test is 59.8%. These findings are in line with the results of earlier research [20]. A review of other PZ-PUKT studies that analyzed the accuracy of the results shows that the total percentage of correct answers ranges from 64.9% to 80.0% [21,22,23,24,25]. Results for the Prevention subscale vary between 68.6% and 77.0% [21,24], while for the Staging subscale, they range from 64.0% [24] to 86.0% [21]. Similarly, on the Wound Description subscale, they range from 56.0% [24] to 77.0% [21]. Compared to these ranges, the results of this study show that the level of knowledge of Croatian nurses is low, with a particularly poor result recorded in the Wound Description and Staging subscales. These findings indicate gaps in knowledge in these key categories and the need for improvement.

Correlation analysis examined the relationship between nurses’ experience and test performance (Prevention, Staging, Wound Description and overall scores). The results show that experience was not significantly correlated with any of the test performance measures. Specifically, experience had a weak negative correlation with Prevention (r = −0.026, *p* = 0.674), Staging (r = −0.068, *p* = 0.268) and overall score (r = −0.034, *p* = 0.578), none of which were statistically significant. Similarly, there was no meaningful relationship between experience and Wound Description percentage (r = 0.009, *p* = 0.885). The obtained coefficients indicate a very weak correlation between the variables, suggesting that experience itself is not a decisive factor in the success of the tested measures. However, the direction and consistency of the results may indicate the existence of subtle relationships that could become more pronounced in a larger sample.

These findings suggest that years of experience do not appear to impact performance on the tested measures, indicating that other factors, such as level of education, may play a more influential role in knowledge and skills related to the assessment areas.

The results of this research are unique compared to those of other studies [26,27], where no statistically significant differences in knowledge were found with respect to work experience.

Regarding test performance, master’s degree holders had the highest mean percentages in Prevention (67.0%), Wound Description (58.6%) and overall score (61.1%), while bachelor’s degree holders had the highest score in Staging (59.0%). One-way ANOVA examined differences in test performance across education levels (technician, bachelor’s, master’s) for Prevention, Staging, Wound Description and overall percentages. No significant differences were found for Prevention (F(2,265) = 0.663, *p* = 0.516) or overall score (F(2,265) = 2.861, *p* = 0.059). However, significant differences were observed for Staging (F(2,265) = 4.098, *p* = 0.018) and Wound Description (F(2,265) = 4.795, *p* = 0.009). Post hoc Tukey’s tests revealed that bachelor’s degree holders scored significantly higher than technicians in Staging percentage (mean difference = 5.11, *p* < 0.05), and master’s degree holders scored significantly higher than technicians in Wound Description percentage (mean difference = 6.81, *p* < 0.05), while no other pairwise differences reached significance. These findings suggest that education level influences performance on Staging and Wound Description tasks, with higher education levels associated with better scores in these domains.

The more influential impact of education on knowledge and education level was observed both in our study and in the majority of studies examining this relationship [28]. However, there is an interesting example of the education model of Slovakian nurses for which the results are exactly the opposite—nurses with a high school level of education showed better knowledge than female nurses with a bachelor’s degree. According to the explanation of the authors, this is a consequence of changes in the education curriculum, which indicates the importance of not only the formal level of education but also the quality of educational programs [26].

One-way ANOVA was conducted to examine differences in test performance percentages across department types (Surgery, Internal, Neurology, ICU and Other). A significant difference was found for Prevention (F(4,263) = 3.17, *p* = 0.014), while no significant differences were found for Staging (F(4,263) = 0.90, *p* = 0.462), Wound Description (F(4,263) = 0.71, *p* = 0.59) or overall score (F(4,263) = 1.73, *p* = 0.14).

Tukey’s post hoc test for Prevention revealed that participants in the Neurology department scored significantly higher than those in the other departments (mean difference = 7.71, *p* = 0.007), while no other comparisons reached statistical significance. These results suggest that department type may influence Prevention, with Neurology department participants demonstrating better performance compared to those in the other departments. However, no significant differences were observed for the remaining test measures. In studies that also employed the PZ-PUKT instrument to assess nurses’ knowledge, it was observed that intensive care unit (ICU) nurses in the United States achieved higher scores on the Staging subscale compared to the Prevention and Wound Description subscales [22], while knowledge about prevention among ICU nurses from three university hospitals in Turkey was insufficient, with an average knowledge score of 43.2 ± 11.4% [29]. Huang and colleagues found that the Staging subscale had the highest scores (97.88%) compared to the other two, with the lowest results in the Prevention subscale (55.66%) [30]. Our study findings show that nurses have a better understanding of prevention compared to staging and wound description, with their understanding of neurology at this hospital being significantly higher than of other clinical specialties. Analyzing their activities, we can conclude that this is a result of continuous education, daily monitoring and mentoring nurses in this department, and the low incidence of PU/PI. Similar results for the prevention scale have been observed in other studies such as the Iranian study by Iranmanesh et al. [31] and the Spanish study by Pancorbo-Hidalgo et al. [32]. This may be because educational programs and hospital policies are primarily focused on preventing pressure injuries. By reviewing the literature to detect contemporary technological developments that contribute to the improvement of educational and clinical practices, we highlight the example of the development of a mobile application based on a contextualized instructional design by Salomé and Ferreira. The application, designed in accordance with the constructivist approach to learning, allows the storage of patient demographic data, assessment of wound characteristics, and identification of risk factors for the development of pressure ulcers. In addition, the application provides personalized recommendations for wound cleansing and selects appropriate therapeutic interventions. This form of digital support represents a valuable tool in clinical practice because it facilitates decision-making, improves pressure ulcer prevention and encourages the implementation of evidence-based nursing interventions [33].

Spearman’s correlation analysis examined the relationships between the recency of attending a lecture (Lecture) and reading about PU/PI (Read) with test performance (Prevention, Staging, Wound Description and overall scores). Both Lecture and Read showed significant positive correlations with Prevention (rho = 0.219, *p* < 0.001 and rho = 0.451, *p* < 0.001, respectively), indicating that more recent exposure to lectures and reading was associated with higher Prevention scores. Similarly, Lecture (rho = 0.256, *p* < 0.001) and Read (rho = 0.328, *p* < 0.001) were significantly correlated with Staging scores, suggesting that those who had attended lectures or read more recently performed better in staging assessments. Read was also significantly correlated with Wound Description (rho = 0.240, *p* < 0.001), while Lecture did not show a significant relationship with this measure (rho = 0.092, *p* = 0.133). Finally, overall scores were significantly associated with both Lecture (rho = 0.243, *p* < 0.001) and Read (rho = 0.434, *p* < 0.001), suggesting that participants who had more recent educational exposure—particularly through reading—tended to perform better overall.

The opposite results about attending lectures or reading an article were found in the previous year in a study by author Fulbrook et al.: neither has a significant impact on the results [23]. A possible explanation for this difference lies in the type and quality of educational content and the manner of their application—if the materials were general or passively presented, their impact on knowledge could be less compared to active information searching via the Internet. Also, the individual motivation of the respondents could play a key role, because those who search for information on their own often show greater interest and engagement in learning. A study by Mohamed et al. highlighted the importance of lifelong continuous education to improve the knowledge and professional practice of nurses [34]. The results of this research conclude that professionally focused educational programs play a key role in improving pressure ulcer prevention, confirming significant progress in the implementation of effective preventive measures after the implementation of a targeted educational program.

The independent samples *t*-test examined differences in test performance percentages between participants who employed the internet for information on pressure ulcers (Yes) and those who did not (No). The results show that Internet users scored significantly higher across all test measures. Specifically, Prevention was significantly higher for internet users (mean = 69.2, SD = 11.17) compared to non-users (mean = 62.5, SD = 12.2), t(266) = 4.7, *p* < 0.001, with a mean difference of 6.72. Similarly, Staging was higher among Internet users (mean = 58.7, SD = 12.66) than non-users (mean = 53.6, SD = 14.0), t(266) = 3.15, *p* = 0.002, with a mean difference of 5.15. Wound Description also showed a significant advantage for Internet users (mean = 58.3, SD = 14.26) over non-users (mean = 51.3, SD = 13.5), t(266) = 4.08, *p* < 0.001, with a mean difference of 6.92. Finally, overall score was higher among Internet users (mean = 63.1, SD = 9.96) compared to non-users (mean = 56.8, SD = 10.7), t(266) = 4.99, *p* < 0.001, with a mean difference of 6.32. These results suggest that using the Internet for information on pressure ulcers is associated with significantly better test performance in all measured categories. Our results are consistent with previous research, which also indicates better outcomes for respondents who actively search for information online or read guidelines [35]. These findings further emphasize the importance of independent access to educational materials in improving knowledge and understanding of key topics.

### 4.1. Strengths

This is the first study on nurses’ knowledge of pressure ulcers/injuries conducted using the PZ-PUKT instrument in Croatia. The strength of this study is that the research population studied consisted of nurses working in all departments that encounter this group of patients, from the Department of Internal Medicine (with subspeciality yard), Department of Surgery (with subspeciality yard), Urology, Orthopedics and Traumatology, Otorhinolaryngology, Psychiatry, Neurology, Infectious Diseases, Dermatology and Venereology, Pediatrics and Department of Anesthesia, Resuscitation and Intensive Care. This enables comprehensive insight into their knowledge and practice, which increases the representativeness and applicability of the results to different clinical environments.

### 4.2. Limitations

A limitation of this study is that it focused solely on knowledge levels, without assessing nurses’ practical skills. This made it impossible to assess the extent to which the acquired knowledge is applied in everyday clinical practice, which is an important direction for future research.

## 5. Conclusions

In this study, we evaluated the primary objective and found that, although the overall level of knowledge among respondents remains suboptimal, the positive influence of formal education and lifelong learning on nurses’ understanding of pressure ulcers is evident. These findings underscore the importance of enhancing and better structuring educational programs, with particular attention to their accessibility, continuity and alignment with clearly defined learning outcomes. The results of this study indicate the need for some revision of the nursing curriculum, with the aim of improving knowledge on the prevention, classification and description of pressure ulcers. Although the findings of this study confirm the connection between increased knowledge and formal education, as well as lifelong learning on this topic, it is necessary to continuously upgrade this knowledge in accordance with the latest scientific findings. In addition, it is important to ensure that these topics are covered in a separate, mandatory course with an appropriate number of hours, which would allow for a deeper understanding and practical application of the acquired competencies. In addition, the need to precisely define thematic units and an appropriate number of teaching hours within the core curriculum for the course is emphasized, in order to ensure uniform knowledge delivery to students. Also, special attention should be paid to practical teaching in the classroom and laboratories, through simulation exercises and clinical practice, in order to improve the skills necessary for the effective treatment of pressure ulcers. Lifelong education on the issue needs to be implemented in an organized and systematic manner through structured educational forms such as lectures and interactive workshops, with clearly defined learning outcomes and their continuous monitoring, with the aim of ensuring the transfer of the latest professional guidelines and scientific knowledge into clinical practice.

In conclusion, the competencies and proactive measures of staff directly contribute to prevention and treatment outcomes, emphasizing the importance of education in improving the quality of healthcare and patient outcomes.

## Figures and Tables

**Table 1:** Basic sociodemographic characteristics of the respondents.

	Technician (n = 113)	Bachelor (n = 102)	Master (n = 53)	Overall (n = 268)
Gender				
Male	5 (4.4%)	2 (2.0%)	0 (0%)	7 (2.6%)
Female	108 (95.6%)	100 (98.0%)	53 (100%)	261 (97.4%)
Age				
Mean (SD)	39.2 (13.0)	35.1 (8.60)	42.8 (8.86)	38.4 (11.0)
Department				
Surgery	49 (43.4%)	21 (20.6%)	7 (13.2%)	77 (28.7%)
Internal	16 (14.2%)	28 (27.5%)	14 (26.4%)	58 (21.6%)
Neurology	18 (15.9%)	22 (21.6%)	16 (30.2%)	56 (20.9%)
ICU	6 (5.3%)	13 (12.7%)	3 (5.7%)	22 (8.2%)
Other	24 (21.2%)	18 (17.6%)	13 (24.5%)	55 (20.5%)
Experience				
Mean (SD)	17.3 (12.6)	12.3 (8.52)	17.7 (10.1)	15.5 (11.0)

**Table 2:** Basic descriptive sample parameters by education level.

	Technician (n = 113)	Bachelor (n = 102)	Master (n = 53)	Overall (n = 268)
Lecture				
<1 year	24 (21.2%)	22 (21.6%)	18 (34.0%)	64 (23.9%)
1–2 years	16 (14.2%)	18 (17.6%)	13 (24.5%)	47 (17.5%)
2–3 years	39 (34.5%)	28 (27.5%)	7 (13.2%)	74 (27.6%)
>4 years	21 (18.6%)	22 (21.6%)	11 (20.8%)	54 (20.1%)
Never	13 (11.5%)	12 (11.8%)	4 (7.5%)	29 (10.8%)
Read				
<1 year	42 (37.2%)	44 (43.1%)	25 (47.2%)	111 (41.4%)
1–2 years	23 (20.4%)	28 (27.5%)	11 (20.8%)	62 (23.1%)
2–3 years	28 (24.8%)	19 (18.6%)	3 (5.7%)	50 (18.7%)
>4 years	14 (12.4%)	10 (9.8%)	12 (22.6%)	36 (13.4%)
Never	6 (5.3%)	1 (1.0%)	2 (3.8%)	9 (3.4%)
Internet				
Yes	42 (37.2%)	62 (60.8%)	25 (47.2%)	129 (48.1%)
No	71 (62.8%)	40 (39.2%)	28 (52.8%)	139 (51.9%)
Prevention (%)				
Mean (SD)	64.8 (12.9)	66.1 (11.0)	67.0 (12.7)	65.7 (12.1)
Wound desc. (%)				
Mean (SD)	51.8 (14.4)	55.8 (13.5)	58.6 (14.5)	54.7 (14.3)
Staging (%)				
Mean (SD)	53.9 (14.8)	59.0 (12.6)	55.0 (11.9)	56.1 (13.6)
Overall (%)				
Mean (SD)	58.0 (11.5)	61.2 (9.81)	61.1 (10.7)	59.8 (10.8)

**Table 3:** One-way ANOVA of test percentages by education level.

Variable	F	df1	df2	*p*
Prevention	0.663	2	265	0.516
Staging	4.098	2	265	0.018 *
Wound Description	4.795	2	265	0.009 *
Overall	2.861	2	265	0.059

Legend: * = statistically significant.

**Table 4:** One-way ANOVA of test percentages by department.

Variable	Department	N	Mean	SD	SE	1-Way ANOVA
Prevention	Surgery	77	64.8	13.66	1.56	F(4,263) = 3.17 *p* = 0.014 *
	Internal	58	66.9	11.54	1.51	
	Neurology	56	69.7	7.50	1.00	
	ICU	22	64.8	11.13	2.37	
	Other	55	62.0	13.70	1.85	
Staging	Surgery	77	56.8	12.27	1.40	F(4,263) = 0.90 *p* = 0.462
	Internal	58	55.5	17.40	2.28	
	Neurology	56	57.7	12.71	1.70	
	ICU	22	57.4	12.68	2.70	
	Other	55	53.3	11.86	1.60	
Wound Description	Surgery	77	54.6	13.32	1.52	F(4,263) = 0.71 *p* = 0.59
	Internal	58	54.6	17.45	2.29	
	Neurology	56	55.0	14.65	1.96	
	ICU	22	58.9	10.68	2.28	
	Other	55	52.8	12.76	1.72	
Overall	Surgery	77	59.7	11.12	1.27	F(4,263) = 1.73 *p* = 0.14
	Internal	58	60.2	12.52	1.64	
	Neurology	56	62.1	8.25	1.10	
	ICU	22	61.0	9.78	2.09	
	Other	55	56.9	10.79	1.46	

Legend: * = statistically significant.

**Table 5:** Spearman’s correlation of Lecture and Read with test percentages.

Variable	Lecture (rho)	Lecture (*p*-Value)	Read (rho)	Read (*p*-Value)
Prevention	0.219 *	<0.001	0.451 *	<0.001
Staging	0.256 *	<0.001	0.328 *	<0.001
Wound Description	0.092	0.133	0.240 *	<0.001
Overall	0.243 *	<0.001	0.434 *	<0.001

Legend: * = statistically significant.

**Table 6:** *t*-test of test percentages by use of internet for PU/PI information (Yes vs. No).

	Yes		No				
Variable	Mean	SD	Mean	SD	t-Value	*p*-Value	Difference
Prevention	69.2	11.17	62.5	12.2	4.7	<0.001 *	6.72
Staging	58.7	12.66	53.6	14.0	3.15	0.002 *	5.15
Wound Description	58.3	14.26	51.3	13.5	4.08	<0.001 *	6.92
Overall	63.1	9.96	56.8	10.7	4.99	<0.001 *	6.32

Legend: * = statistically significant.

## Data Availability

Data collected in the study are not publicly available due to privacy and ethical restrictions but can be made available upon reasonable request from the corresponding author.

## Public Involvement Statement

No public involvement in any aspect of this research.

## Guidelines and Standards Statement

This manuscript was prepared following the procedures outlined in the Strengthening the Reporting of Observational Studies (STROBE). A complete list of reporting guidelines can be accessed via the equator network: https://www.equator-network.org/.

## Abbreviations

The following abbreviations are used in this manuscript:

PU/PI	Pressure ulcer/injury
PZ-PUKT	Pieper-Zulkowski Pressure Ulcer Knowledge Test
ICU	Intensive care unit
